# On the Emission of Urine, as Observed in an Individual Suffering from Ectopia of the Bladder

**Published:** 1850-07

**Authors:** 


*On the Emission of Urine, as observed in an Individual suffering from Ectopia of the Bladder.* By Dr. Parmeggiani.—A man, æt., 30, having entered the hospital at Reggio with ectopia of the posterior wall of the bladder, so as to exhibit the orifice of the ureters very plainly, the opportunity was taken to make some observations upon the mode in which the urine entered the bladder. The statement of physiologists that it passes in, drop by drop, was not found to hold good. On the contrary, from time to time it issued in true jets, sometimes fifteen seconds, sometimes thirty seconds, or even two or three minutes elapsing between these, the time varying according to that which had elapsed since fluids were taken into the stomach. The flow had no dependence upon the circulatory or respiratory movements. When emitted, the urine was fetid and alkaline, but in the act of flowing furnished an acid reaction—which is confirmatory of the statement of Stehberger, that the urine is secreted alkaline and becomes acid in the bladder.

Signs of the presence of iodide of potassium were found in one experiment six minutes, and in another twelve, after it had been swallowed; these increasing during twenty-four hours, and then diminishing until they diappeared.—*Omedei Annali*, vol. cxxiv, p. 241.

